# Associative Interactions among Zinc, Apolipoprotein E, and Amyloid-β in the Amyloid Pathology

**DOI:** 10.3390/ijms21030802

**Published:** 2020-01-25

**Authors:** Shin Bi Oh, Jung Ah Kim, SuJi Park, Joo-Yong Lee

**Affiliations:** 1 Asan Institute for Life Sciences, Asan Medical Center, Seoul 05505, Korea; linzy1@naver.com (S.B.O.); jung.kim0702@gmail.com (J.A.K.); kwissh@naver.com (S.P.); 2 Department of Medical Science, Asan Medical Institute of Convergence Science and Technology, University of Ulsan College of Medicine, Seoul 05505, Korea; 3 Department of Convergence Medicine, University of Ulsan College of Medicine, Seoul 05505, Korea

**Keywords:** zinc, amyloid pathogenesis, apoE/Aβ complexes, proteases, plasmin, Aβ degradation and clearance, chelators

## Abstract

Zinc and apolipoprotein E (apoE) are reportedly involved in the pathology of Alzheimer’s disease. To investigate the associative interaction among zinc, apoE, and amyloid-β (Aβ) and its role in amyloid pathogenesis, we performed various biochemical and immunoreactive analyses using brain tissues of Tg2576 mice and synthetic Aβ and apoE peptides. On amyloid plaques or in brain lysates of Tg2576 mice, apoE and Aβ immunoreactivities increased after zinc chelation and were restored by its subsequent replacement. Zinc depletion dissociated apoE/Aβ complexes or larger-molecular sizes of Aβ oligomers/aggregates into smaller-molecular sizes of apoE and/or Aβ monomers/complexes. In the presence of zinc, synthetic apoE and/or Aβ peptides aggregated into larger-molecular sizes of oligomers or complexes. Endogenous proteases or plasmin in brain lysates degraded apoE and/or Aβ complexes, and their proteolytic activity increased with zinc depletion. These biochemical findings suggest that zinc associates with apoE and Aβ to encourage the formation of apoE/Aβ complexes or large aggregates, raising the deposition of zinc-rich amyloid plaques. In turn, the presence of abundant zinc around and within apoE/Aβ complexes may block the access or activity of Aβ-degrading antibodies or proteases. These results support the plausibility of chelation strategy aiming at reducing amyloid pathology in Alzheimer’s disease.

## 1. Introduction

The main pathological hallmark of Alzheimer’s disease (AD) is the progressive deposition of amyloid-β (Aβ) in the brain. Aβ peptides are formed by sequential cleavage of the amyloid precursor protein (APP) by proteolytic action of β- and γ-secretases [1] and favor spontaneous conformational transition into oligomers and fibrillar aggregates, leading to the formation of amyloid plaques [2]. Familial AD mutations in APP and the Aβ-releasing secretase genes promote the production of Aβ [1].

Aβ peptides are rapidly precipitated with physiological concentrations of zinc in vitro, undergoing conformational changes into oligomers with higher toxicity [3,4]. Zinc binds to Aβ peptides at their N-terminal region that contains one glutamate and three histidine residues, and intramolecular zinc–histidine bridges between adjacent Aβ peptides cause Aβ oligomerization, aggregation, and plaque formation [5,6,7]. Therefore, zinc enrichment is found in amyloid deposits, especially within and around compact core amyloid plaques [8,9]. Metal removal or chelation (using EGTA, N,N,N’,N’-tetrakis(2-pyridylmethyl)ethylenediamine (TPEN), bathocuproine, etc). dissolves or blocks the formation of Aβ aggregates and dissociates Aβ deposits from brain tissue of AD patients [10,11]. Chelating agents such as DP-109, clioquinol (5-chloro-7-iodo-8-hydroxyquinoline) and its derivative PBT2, deferiprone, and deferoxamine reduce Aβ deposition and improve cognitive performance in AD patients and Aβ-transgenic mouse models [12,13,14,15,16,17]. We have demonstrated that a reduction in the cerebral zinc content by genetic ablation of zinc transporter 3 (ZnT3), which controls the sequestration of zinc into synaptic vesicles, attenuates Aβ deposition and cerebral amyloid angiopathy (CAA) in Tg2576 mice overexpressing human APP [18,19]. In turn, accumulating evidence of the causative roles of metals including zinc, copper, and iron in amyloid pathogenesis has recently led to the development of chelating strategies for AD [20,21].

Apolipoprotein E (apoE) is an important regulator of lipid metabolism in the brain, and its polymorphism has been associated with cardiovascular diseases and neurodegenerative diseases. In AD, apoE serves as a pathological chaperone to stimulate Aβ aggregation and fibrillation in amyloid pathogenesis [22,23,24]. Human apoE is a 35 kD glycoprotein composed of 299 amino acids, and the specific structures and functions of the three isoforms of human apoE (apoE2, apoE3, and apoE4) depend on the nature of the amino acids; apoE2 has cysteine residues at positions 112 and 158, whereas the cysteine at position 158 is replaced by arginine in apoE3, and apoE4 contains arginine residues at both positions [24,25]. ApoE binds to Aβ with high avidity in an isoform-specific manner [24,25], resulting in coexistence of these peptides in amyloid plaques [26,27]. ApoE4, the major risk factor of late-onset AD and CAA, binds to and promotes Aβ fibrillation more readily than apoE2 and apoE3 [22,24,28]. A lack of apoE in *hAPP*-overexpressing mice eliminates amyloid plaque deposition in the brain without modulating the level of Aβ [29]. ApoE is also involved in the proteolytic degradation of Aβ by neprilysin or insulin-degrading enzyme in an isoform-dependent manner [30].

As a result, zinc and apoE colocalize with each other and with Aβ in amyloid plaques and blood vessels with CAA [27], where they may be expected to cooperatively induce or maintain Aβ aggregation [31]. As the sulfhydryl groups of cysteine residues are responsible for metal binding [32,33], the arginine substitutions in apoE4 limit its ability to control zinc homeostasis and zinc-dependent molecular events in the AD brain [34,35]. We previously reported that apoE depletion negatively regulates the expression of ZnT3, suggesting that apoE mediates zinc homeostasis in the brain [27]. However, the influence of cooperative interaction between zinc and apoE in amyloid pathology remains largely unexplained.

Here, we provide the hypothesis that zinc might interact with apoE and Aβ to promote their conformational transition into larger apoE/Aβ complexes or aggregates, leading to the deposition of zinc- and apoE-rich compact amyloid plaques resistant to Aβ-degrading proteases or antibodies. Further, we briefly demonstrate the current significance of chelation therapy targeting multiple amyloid pathologies.

## 2. Results

### 2.1. Zinc Chelation Enhances Aβ and apoE Immunoreactivities.

**Figure 1:**
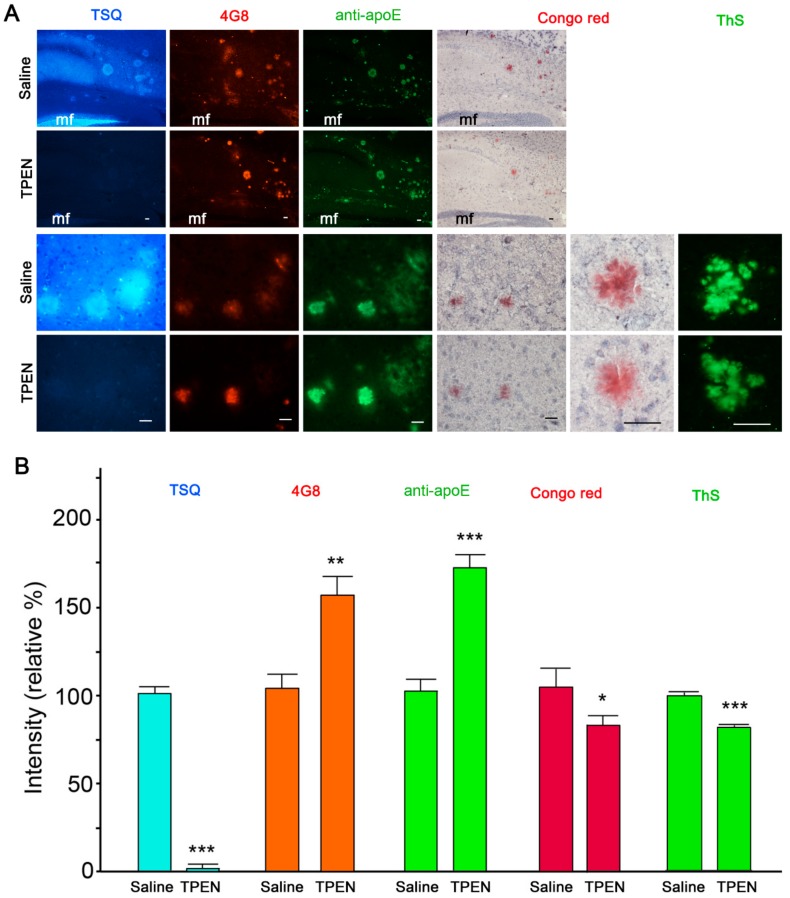
Amyloid-β (Aβ) and apolipoprotein E (apoE) immunoreactivities in amyloid plaques after zinc chelation. (**A**) TSQ staining of zinc (blue in the first column), immunofluorescent staining of Aβ (4G8; red in the second column), apoE (green in the third column), Congo Red (pink in the fourth and fifth columns), and Thioflavin-S (ThS, green in the sixth column) staining of amyloid plaques in the brain of a Tg2576 mouse. The sections were treated with saline (first and third rows) or 100 μM TPEN (second and fourth rows) for 5 min. The magnifications were 100× (first and second rows), 400× (third and fourth rows), and 1000× (fifth and sixth columns), respectively. mf, hippocampal mossy fiber areas. Scale bars, 50 μm. (**B**) Quantification of TSQ-positive zinc levels (blue), 4G8-reactive Aβ (orange), apoE (green), congophilicity (pink), and Thioflavin-S (ThS; green) staining intensity on amyloid plaques shown in (A). The quantitative comparisons were performed using 900 plaques in nine adjacent sections randomly selected from three Tg2576 mice per treatment. * *p* < 0.05, ** *p* < 0.01, and *** *p* < 0.001 by unpaired *t*-test.

Consistent with our previous study [27], immunohistochemistry and zinc-specific 6-methoxy-(8-*p*-toluenesulfonamido)quinoline (TSQ) staining showed complete colocalization of zinc, apoE, and Aβ in compact amyloid plaques in the brain of Tg2576 mouse (Figure 1A). When the sections were pretreated with the zinc chelator TPEN (Kd = 2.6 × 10^−16^ M) [36], TSQ fluorescence completely disappeared from amyloid deposits and the synaptic zinc-rich hippocampal mossy fiber areas (Figure 1). However, when compared with those in saline-treated sections, the intensities of Aβ and apoE immunofluorescence in amyloid plaques markedly increased in TPEN-treated sections (Figure 1). Since protein expression was absent in isolated tissue sections, brighter immunofluorescence indicates increased immunoreactivities of antibodies to Aβ and apoE following zinc chelation. In contrast, congophilicity and thioflavin-S (ThS) fluorescence (measures of the integrity of compact amyloid plaques) were slightly decreased by TPEN treatment (Figure 1).

**Figure 2:**
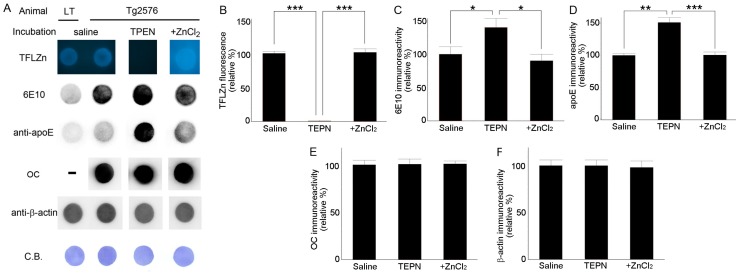
Dot blot analyses of Aβ and apoE in whole brain lysates from Tg2576 mice. Dot blot analyses of Aβ and apoE in whole brain lysates from Tg2576 mice. (**A**) Lysate-loaded (80 μL) membranes were incubated in saline, 100 μM TPEN, or 100 μM TPEN followed by 100 μM ZnCl2, stained with TFLZn, and then reacted with anti-Aβ (6E10 or OC), anti-apoE or anti-β-actin antibody. Coomassie Blue staining (C.B.) was used as a loading control. (**B–F**) Quantitative analyses of results shown in (**A**). Bars denote the percentage of integrated optical density relative to that of the control saline treatment. Measurements were performed in triplicate using brain lysates of the same Tg2576 mouse. * *p* < 0.05, ** *p* < 0.01, and *** *p* < 0.001 by one-way ANOVA.

Thereafter, dot blots were used to evaluate Aβ and apoE immunoreactivities in brain lysates of Tg2576 mouse, which are rich in various forms of Aβ including monomers, oligomers, and fibrils. Lysate-loaded membranes were incubated with TPEN and then again with or without ZnCl_2_ (Figure 2). Here, fluorescence detection of zinc was performed using *N*-(6-methoxy-8-quinolyl)-*p*-carboxybenzoylsulfonamide (TFLZn) rather than TSQ because the latter may also react with lipid components in the brain tissue [8,37]. Treatment with TPEN completely removed zinc from blots, and subsequent ZnCl_2_ supplementation completely restored its levels (Figure 2A,B). In accordance with the above results regarding immunofluorescence staining (Figure 1), the immunoreactivities of 6E10-reactive Aβ (Figure 2A,C) and apoE (Figure 2A,D) were significantly higher in TPEN-treated blots than in saline-treated blots but returned to the initial level after subsequent ZnCl_2_ treatment. However, the immunoreactivities of structure-recognizing antibody OC-reactive Aβ fibrils [38] and β-actin were not affected by TPEN or ZnCl_2_ treatment (Figure 2A,E,F). These findings suggest that zinc binds to Aβ and apoE to deny accessibility or immunoreactivity of antibodies to them.

### 2.2. Zinc Promotes the Aggregations of apoE and/or Aβ Complexes

**Figure 3:**
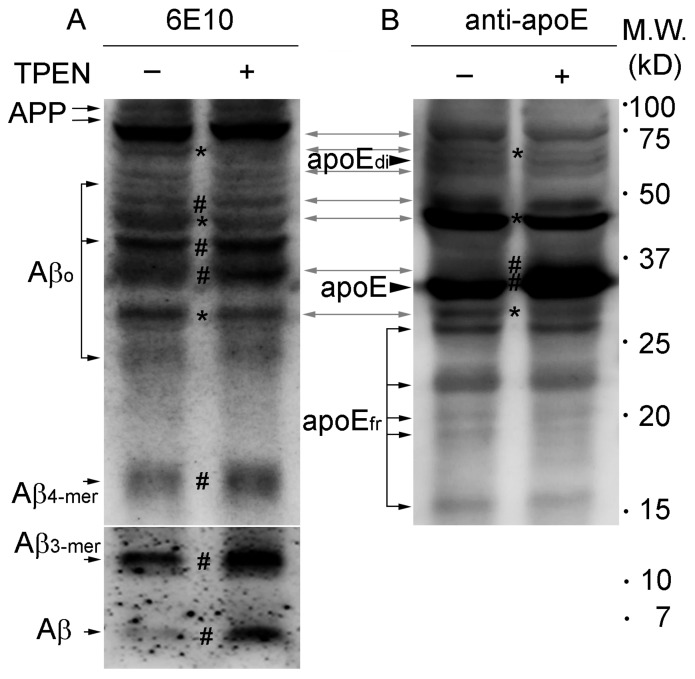
Western blot analyses of Aβ and apoE in whole brain lysates from Tg2576 mice. Protein lysates were prepared with (right lanes) or without (left lanes) 100 μM TPEN, separated on a Tris-Tricine gel, and the transferred blots were incubated with antibody against Aβ (6E10) (**A**) or apoE (**B**). The bidirectional grey arrows represent apoE/Aβ complexes. Asterisks (*) and number signs (#) on blots indicate reduction and increase in band density of proteins by TPEN treatment, respectively. APP, amyloid precursor proteins; Aβo, Aβ oligomers; Aβ4-mer, Aβ quadromers; Aβ3-mer, Aβ trimers; apoEdi, apoE dimers; apoEfr, apoE fragments. Numbers on the right denote molecular weights (kD).

Western blot analysis of brain lysates from Tg2576 mice detected several protein bands for both Aβ and apoE (Figure 3, bidirectional grey arrows) that correspond to apoE/Aβ complexes consisting of various numbers of Aβ and apoE proteins [25,39,40], as well as for either Aβ or apoE (Figure 3, unidirectional black arrows or arrowheads).

When zinc was depleted from lysates using TPEN during homogenization–incubation, decreased densities were noted for some relatively high molecular weight bands (Figure 3, asterisks) corresponding to apoE/Aβ complexes (~45 and ~70 kD) and Aβ oligomers/aggregates (~30 kD), whereas slightly intensified bands (Figure 3, number signs) were observed for small apoE/Aβ complexes (consisting of Aβ monomer and apoE monomer; ~37 and ~47 kD) and Aβ oligomers (~40 kD), quadromers (~16 kD), trimers (~12 kD), and monomers (~4 kD), which lack apoE-binding, as well as for apoE monomers (35 kD). However, it should be here noted that the molecular sizes of the apoE/Aβ complexes and Aβ oligomers/aggregates showing conformational changes upon zinc depletion were different among experiments, in which the downward transition of molecular sizes of the proteins were consistently observed regardless of the different brain samples analyzed.

**Figure 4:**
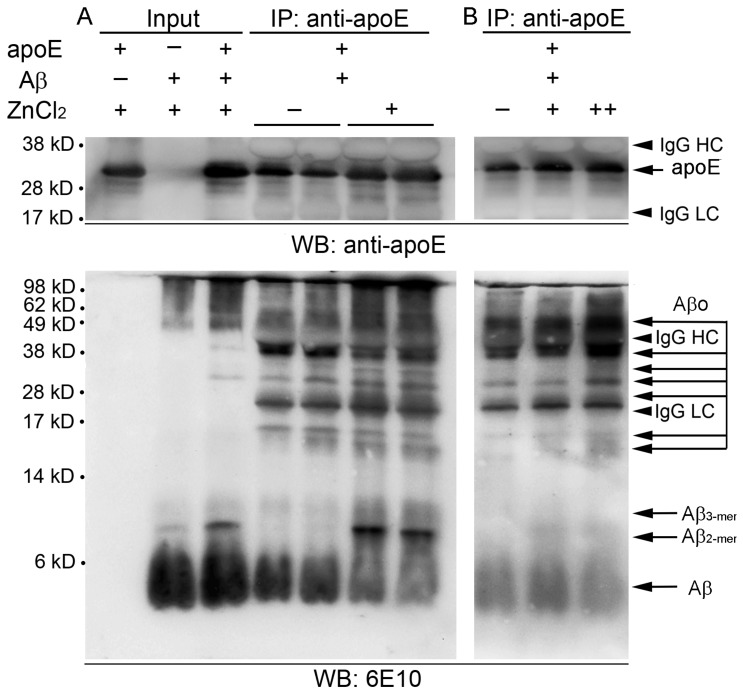
Interactive binding between apoE and Aβ peptides. (**A**) Synthetic apoE3 (5 μg) and Aβ(1-42) (10 μg) peptides were incubated together in zinc-free (-) or zinc-containing (+) buffer (50 μM ZnCl_2_) for 3 h and co-immunoprecipitated with an antibody to apoE. The immunoprecipitates were then subjected to Western blot analysis to probe various forms of apoE (top panels) or Aβ (6E10; bottom panels). (**B**) The co-immunoprecipitation of synthetic apoE3 and Aβ(1–42) peptides was performed in the reaction buffer using different concentrations of ZnCl_2_ (-, 0 μM; +, 10 μM; ++, 50 μM). Comparably, synthetic apoE3 (1.5 μg) and/or Aβ(1–42) (3 μg) peptides were detected as input references for the reaction in zinc-containing buffer (50 μg ZnCl_2_). The dots are representative of three independent experiments. Numbers on the left side show molecular weight (kD). IgG HC, IgG heavy chain; IgG LC, IgG light chain.

To further evaluate the contribution of zinc in assembling the homo- or heteroaggregates of apoE and/or Aβ, after coincubation of synthetic apoE and Aβ(1–42) peptides with or without ZnCl_2_, the mixtures were subjected to co-immunoprecipitation using apoE antibody followed by Western blot analysis with apoE- (top panels in Figure 4) or Aβ-antibody 6E10 (bottom panels in Figure 4). Input mixtures of the two synthetic peptides developed various sizes of 6E10-immunoreactive bands corresponding to Aβ monomers/oligomers/aggregates and apoE/Aβ complexes in the presence of 50 μM ZnCl_2_ (Figure 4A), in a pattern similar to that of mouse brain lysates (shown in Figure 3). We observed that a wide range of Aβ molecules from monomers to oligomers and aggregates, which also looks like those in the input, were co-immunoprecipitated with apoE, indicating direct physical interaction between apoE and Aβ peptides (Figure 4A). The overall level of co-immunoprecipitation of Aβ peptides with apoE antibody was evidently higher with the addition of ZnCl_2_ than that without ZnCl_2_. Upward molecular weight shifts of Aβ were also noticed in zinc-treated immunoprecipitates as relatively lower Aβ monomers (~4 kD) and oligomers (~17 and ~40 kD) were attenuated, whereas higher dimers (~9 kD) and oligomers/aggregates (~25 and >~50 kD) were intensified. Notably, the levels of co-immunoprecipitation with apoE and the conformational shift toward larger sizes of Aβ peptides increased with increasing concentrations of zinc (10–50 μM as ZnCl_2_) in the mixture.

These immunoblotting results support that zinc binding to Aβ and apoE facilitates and stabilizes their aggregation into homo- or heterocomplexes.

### 2.3. Zinc Depletion Encourages Aβ Degradation by Endogenous Proteinases

**Figure 5:**
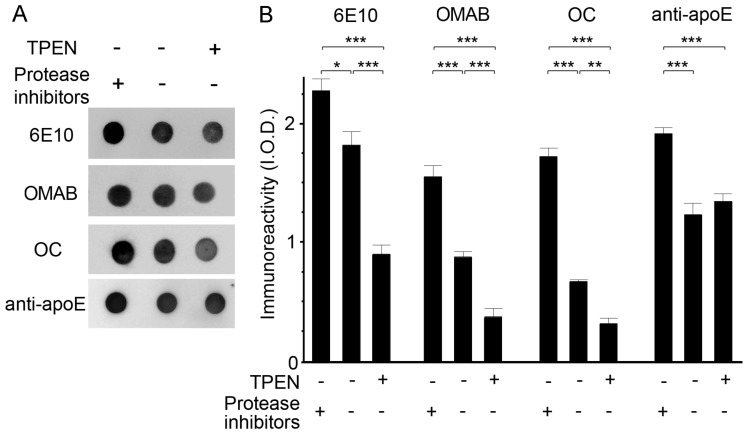
The effects of zinc chelation on endogenous protease-induced apoE/Aβ degradation. (**A**) Representative dot blots of Aβ and apoE in whole brain lysates treated with (+) or without (-) 100 μM TPEN in the presence (+) or absence (-) of EDTA-free protease inhibitors. (**B**) Quantitative analyses of the levels of apoE and total (6E10), oligomeric (OMAB), or fibril (OC) Aβ shown in (**A**). The integrated optical densities (I.O.D.) were arbitrarily measured for the immunoreactivities. Data are from three independent experiments using brain lysates from the same Tg2576 mouse. * *p* < 0.05, ** *p* < 0.01, and *** *p* < 0.001 by one-way ANOVA.

**Figure 6:**
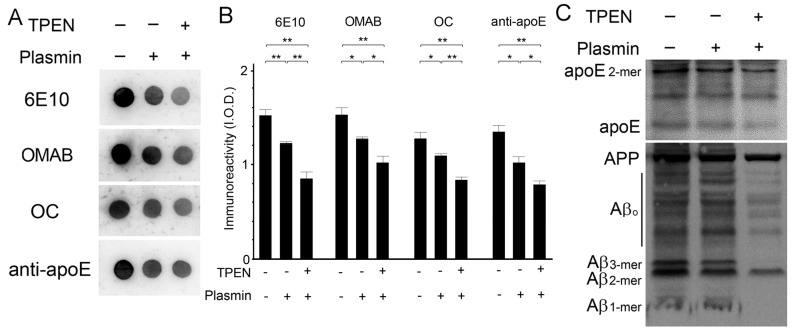
The effects of zinc chelation on plasmin-induced apoE/Aβ degradation. Representative dot (**A**) and Western (**C**) blots for Aβ and apoE of brain lysates prepared in the same volume of buffer after the 30 min incubation with plasmin in the presence (+) or absence (-) of 100 μM TPEN. (**B**) Quantitative measurements of apoE and total (6E10), oligomeric (OMAB), or fibril (OC) Aβ were performed in dot blots from whole brain tissue (**A**). The arbitrary integrated optical densities (I.O.D.) were measured. Data are from three independent experiments using brain lysates from the same Tg2576 mouse. * *p* < 0.05 and ** *p* < 0.01 by one-way ANOVA.

The effect of zinc depletion on the activities of endogenous proteases that degrade Aβ complexes was determined via incubation of brain lysates with TPEN (100 μM) in the presence or absence of protease inhibitors. Dot blot measurements of lysates using antibodies specific for total (6E10), oligomeric (OMAB) [41], or fibrillar (OC) Aβ [38] revealed that protease-mediated degradation of Aβ occurred in protease-inhibitor-free lysates, and was significantly enhanced by the addition of TPEN as the amounts of all Aβ species tested were lower in TPEN-treated lysates than in untreated lysate (Figure 5). By contrast, TPEN had no effect on proteolytic degradation of apoE by endogenous proteases. We also confirmed these findings by using plasmin, a serine protease capable of degrading Aβ [42,43,44], where the proteolytic degradation or clearance of Aβ by plasmin was highest when lysates were incubated with TPEN, as determined by the dot (Figure 6A,B) and Western blot (Figure 6C) assay. It is interesting that the level of apoE was also significantly decreased by the concurrent treatment of plasmin and TPEN. Thus, the total Aβ population, including monomers, oligomers, and fibrillar aggregates, is degraded by active proteases or plasmin, and proteolytic activities and/or accessibilities of proteases to apoE/Aβ complexes could potentially be facilitated by zinc removal from them.

## 3. Discussion

Amyloid plaques might serve as a reservoir of protease-resistant Aβ agglomerates that steadily releases neurotoxic Aβ or delays its clearance [45]. Here, we provided evidence that zinc and apoE may cooperate to develop Aβ aggregates or amyloid plaques and to stabilize them.

We previously found the immunohistochemical colocalization of zinc, apoE, and Aβ in compact amyloid plaques and vasculatures with CAA in the brains of Tg2576 mice [27]. In this study, the immunoreactivities of sequence-specific anti-apoE and anti-Aβ (Aβ17–24-specific 4G8 or Aβ1–16-specific 6E10) antibodies were substantially increased after zinc depletion. In contrast, the immunoreactivity of Aβ fibrils, as detected by dot blotting assays using a conformational structure-specific antibody (Aβ fibrils-specific OC) [38], was not affected. These results imply that zinc may bind to apoE and Aβ to mask the accessibility of sequence-specific antibodies but not structure-specific antibodies.

Cysteine, histidine, aspartate, and glutamate residues represent the majority of zinc-binding residues in proteins [46]. In fact, the zinc-binding property of a protein is determined largely by the sulfhydryl groups of its cysteine residues [32,33]. ApoE3 and apoE2 contain one and two more cysteines than apoE4, respectively [34,35]. The mature human apoE3 protein contains a fairly high percentage (18.1%) of zinc-binding cysteine, histidine, aspartate, and glutamate residues, as well as a metal coordinating four-helix bundle in its N-terminal region [31,35]. A previous study reported that binding of apoE to zinc or other metals modulates lipoprotein oxidation and aggregation of Aβ [31,35]. Together with our immunoreaction experiments, these studies support the idea that direct interactions between zinc and apoE might participate in amyloid pathogenesis.

Consistent with the results of previous studies [25,39,40,47], we showed that monomeric apoE bound to Aβ peptides to form high-molecular-weight apoE/Aβ complexes. When zinc was removed from proteins, high-molecular apoE/Aβ complexes and Aβ oligomers or aggregates decreased markedly, and low-molecular apoE monomers and Aβ species increased. In contrast, coincubation of synthetic apoE and Aβ peptides in the presence of zinc raised their co-immunoprecipitation, creating high-molecular Aβ oligomers and aggregates. Therefore, these data indicate that zinc is capable of encouraging homo- or heterocomplexes of apoE and/or Aβ proteins, and thereafter, the high content of zinc may surround or tighten them into compact amyloid plaques.

Since zinc-rich apoE/Aβ complexes or Aβ aggregates lowered their immunoreactivity with apoE- or Aβ-specific antibody, it is of concern whether they also influence the activities of Aβ-degrading proteases [48]. We tested this hypothesis by evaluating the activity of endogenous proteases to degrade Aβ proteins in zinc-removed brain lysates. When proteases remained active by eliminating protease inhibitors in the preparation of the lysate, pretreatment of TPEN consequently reduced the amount of total Aβ, including oligomeric and fibril Aβ, as compared to untreated controls. Moreover, an incubation with plasmin, a serine protease with broad substrate specificity toward extracellular protein components containing Aβ deposits [42,43,44], also resulted in the considerable reduction of Aβ proteins in TPEN-treated lysates. These in vitro results suggest that zinc binding to or surrounding apoE/Aβ complexes or Aβ aggregates could protect them from proteolytic degradation and that zinc depletion could raise the activity of proteases to degrade Aβ.

Despite recent unfortunate failures of Aβ-targeting drugs, reduction in Aβ accumulation is still considered a potential therapeutic target for AD [49,50,51]. Since soluble Aβ oligomers represent the primary neurotoxic species, which may be steadily released from the higher-ordered Aβ assemblies such as apoE/Aβ aggregates and amyloid plaques [45,52], zinc participating in their production has also been noted to be an alternative target for treating amyloid pathology [14,15,16,17]. Furthermore, an approach that requires exploration is the improvement of the degradation and clearance of Aβ with Aβ-degrading antibodies or proteases [48,49,50,53]. We previously showed the degradative actions of the plasmin proteolytic system toward Aβ proteins and amyloid pathology [54]. Interestingly, zinc-binding provides rigidity and denies access of proteolytic enzymes to apoE/Aβ aggregates [55], rendering them resistant against Aβ-degrading antibodies or proteases. In previous reports, it was shown that zinc can directly inhibit the activity of proteases such as serine proteases [56,57], which contain tissue-type plasminogen activator (tPA), plasmin [58,59], and kallikreins [60] with a high affinity for zinc, differently from metallopeptidases that require zinc for their proteolytic activity (e.g., neprilysin, insulin-degrading enzyme (IDE), and angiotensin-converting enzyme) [48]. Therefore, zinc enriched around apoE/Aβ aggregates or amyloid plaques [8,9] may be enough to inhibit Aβ-degrading activity of serine proteases as depicted in this study. Further, because apoE binds with Aβ to facilitate its degradation by proteolytic enzymes such as neprilysin and IDE [30], zinc-mediated apoE-Aβ-binding may also influence Aβ degradation.

While this study solely describes the involvement of zinc in Aβ aggregation, a large body of studies has implicated metals such as copper, iron, and aluminum as well as zinc in Aβ-induced oxidative, neuroinflammatory, and neurodegenerative processes, and thus their intercepting chelators have emerged for treatment of AD pathology [20,21,61,62]. Indeed, several chelating drugs have been recently tested in animal or human AD trials, in which deferoxamine, pyrrolidine dithiocarbamate (PDTC), clioquinol derivatives, and some lipophilic chelators (e.g., DP-109) had notable success in reducing amyloid pathology [12,13,14,15,16,17,63]. We also identified two small molecules that hinder the metal-mediating Aβ oligomerization and attenuate metal-Aβ-induced oxidation and toxicity, most importantly reducing amyloid pathology and improving cognitive deficits in a mouse model of AD [64,65]. Although there was a disappointing phase 2 result for a chelating drug PBT2, these metal-chelating agents have received attention because they can readily penetrate the brain–blood barrier (BBB) to significantly modulate amyloid pathology in the brain. Further development of BBB-permeant chelators will show promise for treating a variety of amyloid pathogenesis in AD.

In conclusion, our study suggests the bifunctional effect of zinc manipulation on amyloid pathology, which may modulate the generation and degradative clearance of toxic Aβ, respectively. It should be noteworthy that combined application of zinc modulator (such as deferiprone, deferoxamine, clioquinol, and its derivative PBT2, or DP-109) [12,13,14,15,16,17] and Aβ-degrading antibody or protease may be a potential therapeutic alternative to reduce amyloid pathology. Finally, this study, taken together with previous reports of the multifaceted roles of zinc in the amyloid pathology [20,21], supports chelation therapy as a potential treatment for AD.

## 4. Materials and Methods

### 4.1. Animal Study

Animal studies were performed under an IACUC-approved protocol and in accordance with the Guideline for Laboratory Animal Care and Use of the Asan Institute for Life Sciences, Asan Medical Center (Seoul, Korea). Brain tissue was collected from 18-month-old female Tg2576 transgenic mice expressing the human Swedish double mutant (K670N/M671L) APP_695_ protein [66]. The mice had ad libitum access to food and water under a 12 h light/12 h dark cycle.

### 4.2. Tissue Preparations

The right brain hemisphere of each mouse was snap-frozen in liquid nitrogen for immunoblotting analyses. For histological analyses, sagittal sections of the left hemisphere were prepared on 1% poly-l-lysine-coated glass slides using a cryostat (HM550; Microm, Walldorf, Germany).

### 4.3. Detection of Amyloid Plaques

Brain sections were stained with hematoxylin (Gill type III; Merck, Darmstadt, Germany) and then with Accustain^®^ Congo Red amyloid staining solution (Sigma, St. Louis, MO, USA). Sections were examined under a light microscope (Eclipse 80i; Nikon, Tokyo, Japan). The sections were also stained with 1% thioflavin-S (ThS; Sigma) in 50% ethanol in the dark and examined under the fluorescence microscope (Eclipse 80i).

### 4.4. Immunohistochemistry

Mouse antihuman Aβ(17–24) (4G8, 1:1000 dilution; BioLegend, San Diego, CA, USA) and goat antimouse apoE (M-20, 1:200 dilution; Santa Cruz, Delaware, CA, USA) antibodies were used. Brain sections underwent fixation with 4% paraformaldehyde in phosphate-buffered saline (PBS; pH 7.2) and were briefly treated with 70% formic acid (Sigma), blocked with 3% normal donkey serum (Vector Laboratories, Burlingame, CA, USA) and 0.3% Triton X-100 (Sigma) in PBS, and subsequently incubated with the primary antibody followed by Alexa Fluor 488- or 555-conjugated secondary antibody (1:1000 dilution; Invitrogen, Carlsbad, CA, USA).

### 4.5. Fluorescent Zinc Staining

Unfixed brain sections were wet with physiological saline (0.9% NaCl, pH 7.2) and then stained with 4.5 µM *N*-(6-methoxy-8-quinolyl)-*p*-toluenesulfonamide (TSQ; Invitrogen) in a buffer containing 140 mM sodium barbital and 140 mM sodium acetate (pH 10.0) for 90 s [8,9]. After briefly washing in saline, sections were examined under a fluorescence microscope with a UV-2A filter (dichroic, 400 nm; excitation, 330–380 nm; barrier, 420 nm) (Eclipse 80i).

To evaluate the zinc level in brain lysates from Tg2576 mice, lysate-loaded dot blots were air-dried, stained with 0.1 mM N-(6-methoxy-8-quinolyl)-*p*-carboxybenzoylsulfonamide (TFLZn; Sigma) in Tris buffer (pH 8.0) [8], and then examined under a fluorescence microscope with a UV-2A filter.

### 4.6. Immunoblot Analysis

All solutions and buffers used were treated with Chelex-100 resin (Bio-Rad, Hercules, CA, USA).

Whole brain hemispheres were homogenized in PBS (pH 7.4) with or without EDTA-free Protease Inhibitor Cocktail (Roche, Indianapolis, IN, USA), and the lysates were collected by centrifugation. The amount of protein in the lysate was measured using a bicinchoninic acid assay (Bio-Rad).

For dot blotting, lysates containing protein (5 μg) were dropped on methanol-wet Immobilon-PSQ polyvinylidene difluoride (PVDF) membranes (Millipore, Billerica, MA, USA) and air-dried. For western blot analyses, proteins were dissolved in sample buffer (200 mM Tris-HCl (pH 6.8), 40% glycerol, 2% sodium dodecyl sulfate (SDS), and 0.04% Coomassie Blue G-250) without dithiothreitol or β-mercaptoethanol and subsequently separated on 12% or 16.5% Mini-PROTEAN^®^ Tris-Tricine Precast Gels (Bio-Rad) under nonreducing conditions. Proteins were transferred onto PVDF membranes using a semidry blotter (TE70 PWR; Amersham Biosciences, Uppsala Sweden). After blocking with 5% skimmed milk and 1% bovine serum albumin (Bovogen, Melbourne, Australia) in TBS-T buffer, the dot and Western blots were reacted with the primary antibody (anti-Aβ(1–16) (6E10, 1:1000 dilution; BioLegend), anti-Aβ oligomers (OMAB) (1:800; Agrisera, Vännäs, Sweden), anti-Aβ fibrils (OC; 1:250; Millipore), anti-apoE (AB947, 1:2,000; Chemicon, Temecula, CA), or anti-β-actin (1:500; Sigma)), and then with horseradish peroxidase-conjugated secondary antibody (1:5000; Santa Cruz). Immunoreactive proteins were visualized using Immobilon Western Chemiluminescent HRP Substrate (Millipore) and the Davinch-Chemi^®^ Chemiluminescence Imaging System (CAS-400SM; CoreBio, Seoul, Korea). Band intensities were measured using ImageJ software (National Institutes of Health, Bethesda, MD).

### 4.7. Co-immunoprecipitation

To detect the interaction between apoE and Aβ, synthetic apoE3 (BioVision, Milpitas, CA, USA) and Aβ(1–42) (Bachem, Bubendorf, Switzerland) peptides were incubated together in PBS (pH 7.4; 25 μL) with or without ZnCl_2_ at 37 °C. Following immunoreaction with anti-apoE antibody (AB947; Chemicon), Protein G-Sepharose beads (GE Healthcare, Buckinghamshire, UK) were added, and the reaction mixture was further incubated. The immunoprecipitate beads were washed in PBS and dissociated in tricine sample buffer (200 mM Tris-HCl (pH 6.8), 40% glycerol, 2% SDS, and 0.04% Coomassie Blue G-250) and centrifuged to obtain supernatant protein samples, which in turn were separated by electrophoresis on Tris-Tricine Precast Gel (Bio-Rad) under nonreducing conditions. Proteins were visualized by Western blot using anti-apoE (AB947; Chemicon) or anti-Aβ (6E10; BioLegend) antibody.

### 4.8. Proteolytic Degradation of apoE/Aβ Complexes by Endogenous Proteases or Plasmin

In order to evaluate the degradation of apoE/Aβ complexes by endogenous proteases, the brain lysates of Tg2576 mice were homogenized and incubated for 30 min in PBS (pH 7.4) with or without EDTA-free Protease Inhibitor Cocktail (Roche) and 100 μM N,N,N′,N′-tetrakis(2-pyridylmethyl)ethylenediamine (TPEN; Sigma).

The plasmin-induced proteolytic degradation of apoE/Aβ complexes was tested as previously described [42,43] with some modification for the tissue sample. Briefly, brain lysates were prepared in PBS (pH 7.4) with or without 100 μM TPEN by homogenization and centrifugation. The protein pellets were resuspended and incubated at 37 °C in a reaction buffer (25 μL; 100 mM Tris, pH 7.4, 0.1% Tween 20, and 0.1 mM EDTA) with purified human plasmin (50 μg; Innovative Research, Novi, MI, USA) [42,43]. Thereafter, the reaction was halted by centrifugation, and the pellets were dissolved in sample buffer followed by dot or Western blot analysis for quantification of Aβ and apoE proteins.

### 4.9. Statistics

Data are presented as the mean ± SEM. Statistical comparisons were performed by unpaired Student’s *t* test or one-way ANOVA with Newman-Keuls post hoc test, and a value of *p* < 0.05 was considered significant.
